# RPL24 as a potential prognostic biomarker for cervical cancer treated by Cisplatin and concurrent chemoradiotherapy

**DOI:** 10.3389/fonc.2023.1131803

**Published:** 2023-10-18

**Authors:** Cheng Ming, Xuelian Bai, Lifeng Zhao, Dedong Yu, Xiaomin Wang, Yun Wu

**Affiliations:** ^1^Department of Oncology, Baotou Central Hospital, Inner Mongolia Medical University, Baotou, China; ^2^Department of Oncology, Baotou Central Hospital, Baotou, China; ^3^Institute of Translational Medicine, Baotou Central Hospital, Baotou, China

**Keywords:** cervical cancer, RPL24, concurrent chemoradiotherapy, prognosis, biomarker

## Abstract

Cervical carcinoma (CC) is the one of most common gynecologic cancers worldwide. The ribosomal proteins (RPs) are essential for ribosome assembly and function, and it has been verified that the abnormal expression of RPs was closely associated with tumorigenesis. In this study, we found that the RP large subunit 24 (RPL24) expression level was upregulated after the CC cell lines SiHa and HeLa were treated with Cisplatin (CDDP) *in vitro*. Simultaneously, a nude mouse xenograft model was used to examine the effect of RPL24 on tumor growth *in vivo*, which showed that overexpression of RPL24 can suppress tumor growth. Furthermore, we proved that RPL24 expression increased after CC patients were treated with concurrent chemoradiotherapy (CCRT), and the higher expression of RPL24 predicted a better prognosis using clinical data from 40 CC patients, verified via the Kaplan-Meier Plotter and LOGpc. These results revealed that RPL24 can be considered a potential biomarker to predict the prognosis of CC patients and assess CCRT efficacy.

## Introduction

Cervical cancer (CC) is one of the most common types of carcinomas among women globally, after only breast, colorectal, and lung cancer (1). Thus, identification of prognostic biomarkers or therapeutic targets for the management of CC is strongly warranted. The ribosomal proteins (RPs) are essential for ribosome assembly and function, including extra-ribosomal functions such as activation of p53-dependent or -independent pathways in response to large numbers of extracellular or intracellular stimuli and stress, resulting in cell cycle arrest, senescence, and apoptosis (2). Rpl24 encodes a highly basic protein of 157 amino acids. As a member of the RP family, Ribosomal protein L24 (RPL24) is a component of the 60S large ribosomal subunit and plays an essential role in ribosome biogenesis.

Large bodies of evidence have demonstrated that the abnormal expression of RPs, such as up- or down-regulation, is closely associated with tumorigenesis in different types of cancers, and high expression of RPs is a prognostic factor in some kinds of tumors (3). Abnormal RP expression has been verified in diverse cancer types in recent years using high-throughput techniques (4–7). To date, little is known about the role of RPL24 in CC. The extra-ribosomal functions of RPs are closely related to oncogenesis in different cancers. It has been reported that when RP is deleted or reduced, ribosome biogenesis is blocked, and ribosome biogenesis has recently emerged as an effective target for cancer therapy (8). Recently, with increased understanding of the RP-MDM2-p53 pathway, some RPs are known to block MDM2-mediated p53 ubiquitination and degradation by inhibiting MDM2 activation, thereby affecting cell cycle progression and apoptosis (9). Previous studies have shown that some RPs, including RPL5, RPL11, RPL23, RPL26, RPS2, RPS7, RPS14, RPS25, and RPS26, play critical roles in regulating p53 by interacting with MDM2 (10, 11).

The Belly Spot and Tail (Bst) mouse phenotype is caused by mutations of RPL24, resulting in defects of the eye, skeleton, and coat pigmentation (12, 13). The phenotype of these mice is largely caused by the aberrant upregulation of p53 protein expression during embryonic development. It has been proposed that RPL24 deficiency triggers the p53 response (14). *Rpl24^Bst^* mutant mouse also has been used to suppress protein translation and limit tumorigenesis in multiple mouse models of cancer (15). Adult hematopoietic stem cells (HSCs) self-renewal requires precise control of protein synthesis, an increase or decrease in which can impair HSCs function and may lead to leukemia or bone marrow (BM) failure (16). Ribosomal protein L24 mutation (Rpl24^Bst/+^) is a ribosomal mutation that impairs ribosomal biogenesis, resulting in reduced Rpl24^Bst/+^ HSCs protein synthesis and thus disrupts both fetal and adult HSCs self-renewal. Recent studies show that *Pten* deletion increased protein synthesis while *Rpl24Bst/+* blocked this effect, restoring HSC function and delaying leukaemogenesis in *Rpl24Bst/+* mice (17). RPL24 depletion suppresses tumorigenesis, proliferation and extends survival by promoting eEF2 phosphorylation via eEF2K in pre-clinical mouse model of colorectal cancer (CRC) with *Apc* deletion and *Kras* mutations (15). RPL24 recombinant protein has a significant tumor-suppressor effect in tumor-bearing mice and the human hepatocellular carcinoma HepG2 cell line (18). However, this corresponding mechanism study demonstrated that partial loss of RPL24 can inhibit the tumorigenesis mediated by the Akt or Myc-driven genes via translational inhibition of a subset of cap (eIF4E)-dependently translated mRNAs. Moreover, HDAC inhibitors used as conventional epigenetic drugs for tumor treatment, such as trichostatin-A (TSA), can inhibit the malignant proliferation of tumors through downregulation of RPL24 expression by inducing acetylation (19). In addition, RPL24 overexpression may confer some amycin resistance in the human hepatocellular carcinoma HepG2 cell line, according to the same research findings (20). Therefore, the role and significance of RPL24 in liver cancer appear contradictory. To date, little is known about the role of RPL24 in CC and the role of RPL24 in the effect appraisal of concurrent chemoradiotherapy (CCRT) of CC remains understudied. The research provides a solid basis for the elucidation of the RPL24’s mechanism in CCRT of CC and a meaningful target for its treatment and prognosis.

## Materials and methods

### Bioinformatics

Based on The Cancer Genome Atlas (TCGA) database, the differential expression of RPL24 in CC tissues and paracancerous tissues was analyzed. The related genes that were positively and negatively related to the expression of CC were downloaded from TCGA database, and these genes were imported into Metascape bioinformatics analysis website tools (https://metascape.org/gp/index.html#/main/step1) for KEGG enrichment analysis to analyze the possible regulatory mechanism between RPL24 and cell cycle in CC. To detect the correlation between RPL24 level and recurrence-free survival (RFS), overall survival (OS), and progression-free survival (PFS) in CC patients, the K-M Plotter was adopted to perform a survival curve analysis with the Kaplan-Meier Plotter (https://kmplot.com/analysis/) and LOGpc (https://bioinfo.henu.edu.cn/DatabaseList.jsp).

### Cell culture and *in vitro* treatments

The human CC cell lines SiHa and HeLa were obtained from ATCC (Manassas, VA, USA) and cultured according to the manufacturer’s instructions. These cell lines were plated into 6-well culture plates and were incubated at 37°C in a humidified atmosphere containing 95% air and 5% CO_2_. After 24 h of culture, the cells adhered to the wall (approximately 3×10^6^ cells) and treated with 20 uM CDDP (Catalog No.P4394-250MG, Sigma, USA), which was dissolved in normal saline before use. After being treated for 48 hours, the cultured cells were harvested.

### Flow cytometry assay

Flow cytometry is a valuable tool for determining the percentage of cells in each phase of the cell cycle (G0/G1, S, and G2/M) *in vitro* experiment, flow cytometry was used to detect changes in the cell cycles of HeLa and SiHa cells at different timepoints before and after treatment with CDDP(cisplatin). The cells were trypsinized and washed with PBS, followed by fixation at -20°Cwith 70% cold ethanol and permeabilization with 0.2% Triton-X100. After removing the supernatant, cells were stained with 500 μl propyl iodide staining solution to determine the DNA content of cells. The cells were incubated at 37°C for 20 minutes and the cell cycle was detected by flow cytometry. Flowjo software (Home | FlowJo, LLC) was used to simulate cell cycle distribution for correlation cycle fitting, and determine the proportion of cells in G0/G1, S, and G2/M phases.

### Western blotting

Western blotting was used to detect the expression levels of RPL24 protein in SiHa and HeLa before and after CDDP treatment. Equal amounts of protein samples were extracted from cells with RIPA lysis buffer. Then 50 µg of protein were loaded from each sample and separated using 10% SDS-PAGE before being electrophoretically transferred to PVDF membranes. 5% non-fat dry milk skimmed milk powder was dissolved in PBST(0.1% Tween 20) and blocked with PVDF membrane for 1 hour at room temperature.

Western blot with RPL24 antibody (Cat No: 17082-1-AP, Proteintech Co.,LTD, CN) at dilution of 1:500 incubated at room temperature for 1.5 hours. The membranes were washed with tris-buffered saline (TBS) and exposed to primary antibodies (RPL24 antibodies) at 4°C. Then the membranes were washed with TBST and incubated with appropriate a second antibody conjugated to horseradish peroxidase for 2 hours at room temperature. Eventually, the proteins expression of interest were visualized using an enhanced chemiluminescence reagent (Enlight Buffer from Engreen Co.,Ltd, CAT# No.29050, CN). The results of Western blotting were quantified using the semi-quantitative software ImageJ, the ratio of RPL24[at 1:600 dilution Wuhan sanying(Rabbit Polyclonal antibody)]/CCNB1[at 1:800 dilution AF6627(Rabbit Polyclonal antibody)]/p53[at 1:800 dilution AP062 (Murine monoclonal antibody)] to β-actin[at 1:1500 dilution AP2811 (Murine monoclonal antibody)] was subsequently calculated.

### Vector construction and transfection

To overexpress RPL24, we cloned full-length homo sapiens RPL24 sequences (NM_000986.4) into the vector pcDNA3.0. The RPL24 overexpression plasmid and empty plasmid were respectively transfected into SiHa cells using the Lipofectamine 2000 reagent (Invitrogen) according to the manufacturer’s protocol. The positive clones, which were SiHa cells with a high expression of RPL24, were screened using a medium containing G418 (Calbiochem) and then the efficiency of transfection was examined using the western blot test. To obtain cell lines stably overexpressing RPL24, RPL24 was cloned into the lentivirus plasmid Pez-Lv105. Recombinant lentivirus infection and screening were performed according to the manufacturer’s protocol (GeneCopoeia, Rockville, MD, USA).

### Construction of a nude mice subcutaneous transplantation model

All animal experiments in this study were agreed on by the Guide for the Care and Use of Laboratory Animals. Ten 6-week-old, female NOD/SCID mice (SHANGHAI SLAC, Shanghai) were purchased, housed in the animal facility at the Translational Medicine Central Laboratory, and randomly assigned to one of two groups. The mice were raised under standard conditions (12-hour light and dark cycle, 22 ± 0.5°C, and relative humidity of 40% to 70%, with ad libitum access to food and water).

Stable cell lines were obtained using screening with puromycin. These cell lines from the control and interference groups, which were transfected with RPL24, were formulated as a single-cell suspension of cells and injected into the subcutaneous skin of nude mice at 0.2 ml (about 3×10^6^ cells per side). After a single subcutaneous injection of RPL24 cells into immunodeficient NOD/SCID mice, tumor formation and enlargement were observed 7-9 days later until the tumor size was significantly different, conforming to welfare ethical review of laboratory animal (tumor diameter should not be large, generally no larger than 15mm). The grafts appeared 21 days later. The tumor-bearing mice were sacrificed by dislocation, and the xenografts were dissected. Before the mice were sacrificed, they were euthanized with CO_2_ inhalation to alleviate pain. No mice were treated with drugs while their general status and tumor volume were observed after tumorigenesis. We calculated the volume of subcutaneous tumors in nude mice regularly every 5 days and plotted the volume change curve of subcutaneous tumors. Tumor volume analyses were based on the following equation: Volume =(length ×width^2^)×1/2. At the end of the study, tumor weight was measured.

### Patients and treatment

Data from 40 patients hospitalized with newly diagnosed CC from January 2017 to December 2019 in Baotou Central Hospital were collected and analyzed. The therapeutic methods were pelvic field ± para-aortic extended field radiotherapy, accompanied by concurrent platinum-based chemotherapy.

The inclusion criteria were histopathologic diagnosis of cervical squamous cell CC with complete clinical data, International Federation of Gynecology and Obstetrics (FIGO) stage IB1-IVA, KPS>70, agreement to receive CCRT, acceptance of a cervical puncture biopsy before and after CCRT, and signed voluntary informed consent. All 40 patients underwent the treatment as planned and were examined regularly. This study was approved by the Ethics Committee of Baotou Central Hospital.

### Immunohistochemical analysis

CC tissues were obtained, fixed in 10% formalin, embedded in 5μm- thick paraffin sections. Sections with well-preserved morphology were used for immunohistochemical staining. After dewaxing and hydration series, 17082-1-AP (RPL24 antibody, Proteintech Co.,LTD, CN) at dilution of 1:50 prepared in PBS buffer was incubated at room temperature for 2 hours, followed by immunohistochemistry kit [purchased from DAKO Co.,LTD, Denmark (Cat#:GK500705)] staining. The result was determined by the percentage of positive stained cells and cell staining intensity as follows: negative −, weak positive +, positive ++, and strongly positive +++. To eliminate scoring errors, two researchers independently reviewed each tissue section.

The results were analyzed to determine the pathological pattern and malignancy grade. Then the RPL24 expression of CC tissues after CCRT was detected, and the clinical stage, age, effect assessment of CCRT, apparent diffusion coefficient (ADC) value, and elated clinical factors were combined for evaluation.

### Statistical analysis

All experiments were independently performed at least three times. The influence of RPL24 expression on the survival outcome of patients with CC was analyzed using the Kaplan-Meier method using online public databases. Differences between groups were performed using unpaired t-tests, All the data were represented as mean ± standard deviation(SD) from three independent experiments. Fisher’s exact probability test was used to process the count data. The correlation of RPL24 expression before and after CCRT was analyzed using the Spearman test. Statistical analysis was performed using SPSS version 26.0 software and GraphPad Prism v9.4.1. *P*<0.05 was considered as statistically significant.

## Results

### RPL24 expression was down-regulated in CC

**Figure 1:**
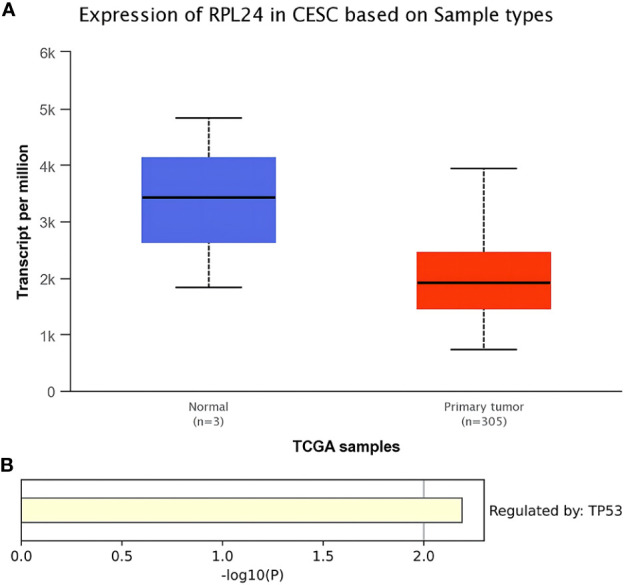
Bioinformatics analysis of RPL24 in CC. **(A)** Expression of RPL24 in CC tissues in TCGA database (r<0.05). **(B)** Enrichment analysis of RPL24 positively related genes in CC in Transcriptional Regulatory Relationships Unraveled by Sentence-based Text mining (TRRUST) database.

TCGA database analysis showed that the expression level of RPL24 in CC tissues was reduced comparing with normal tissues (**Figure 1A**) (P<0.01). In addition, KEGG enrichment analysis revealed that the regulation of RPL24 was related to p53 in CC (**Figure 1B**).

### High RPL24 expression predicts favorable prognosis in CC

**Figure 2:**
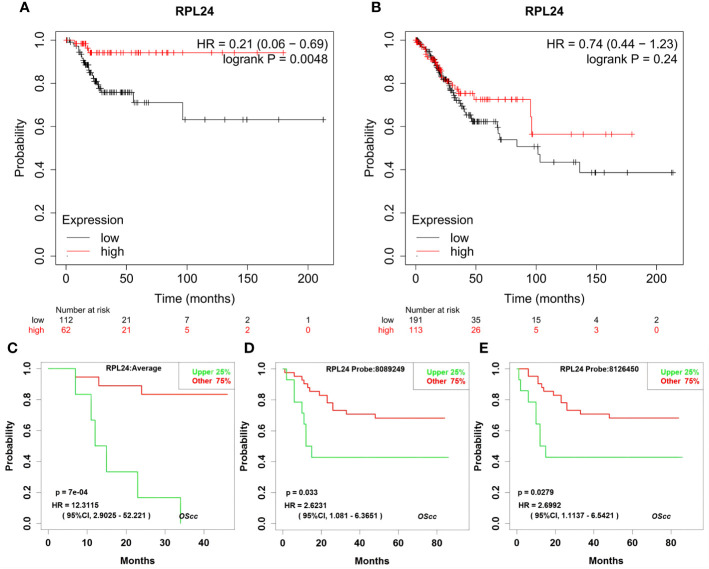
Effects of RPL24 on survival of patients with cervical cancer. The influence of overexpressed RPL24 on the survival of CC patients. **(A, B)** The RFS and OS of patients with cervical cancer related to RPL24 was acquired from the Kaplan online database. **(C)** Correlation between RPL24 expression and OS in GSE14404 dataset. **(D, E)** Correlation between RPL24 expression and PFS in GSE52904 dataset. CI, confidence interval; HR, hazard ratio; RPL24, ribosomal protein large subunit 24; OS, overall survival; PFS, progression- free survival; RFS, recurrence-free survival.

The prognostic value of RPL24 in CC patients was tested using the K-M Plotter database and KaplanMeier Plotter Analysis of LOGpc. According to the Kaplan-Meier database, high expression of RPL24 was related to a favorable RFS in CC patients (HR=0.21; 95% CI, 0.06~0.69, *p*=0.0048) (**Figure 2A**) but was not correlated with OS (*P*>0.05) (**Figure 2B**). Meanwhile, an analysis based on the GSE14404 dataset in LOGpc showed that the high-RPL24 expression group had favorable OS (HR, 12.3115; 95%CI, 2.9025~52.221, *P*=7e-04; **Figure 2C**) for CC patients. In addition, an analysis of the GSE52904 dataset demonstrated that the increased-RPL24 expression group had favorable PFS (respectively, HR, 2.6231; 95%CI, 1.081~6.3651, *P*=0.033; HR, 2.6992; 95%CI, 1.1137~6.5421, *P*=0.0279; **Figures 2D, E**) for CC patients. These results indicate that the RPL24 expression level has prognostic significance in the GEO database and that CC patients with high RPL24 expression often have favorable prognoses. In conclusion, high RPL24 expression was significantly correlated with favorable RFS, OS, and PFS in CC patients.

### RPL24 expression increased by blocking cell cycle after CDDP treatment *in vitro*

**Figure 3:**
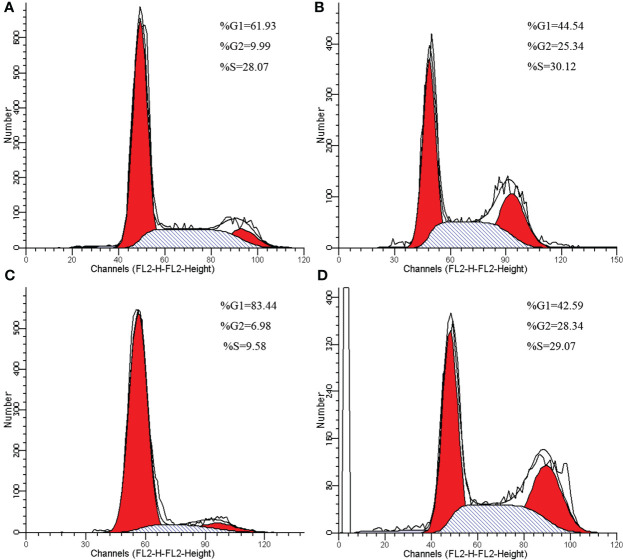
Cell cycle changes of HeLa and SiHa cells after CDDP treatment. **(A)** Cell cycle of HeLa cells in control group. **(B)** Effects of CDDP treatment on cell cycle of HeLa cells. **(C)** Cell cycle of SiHa cells in control group. **(D)** Effects of CDDP treatment on cell cycle of SiHa cells.

**Figure 4:**
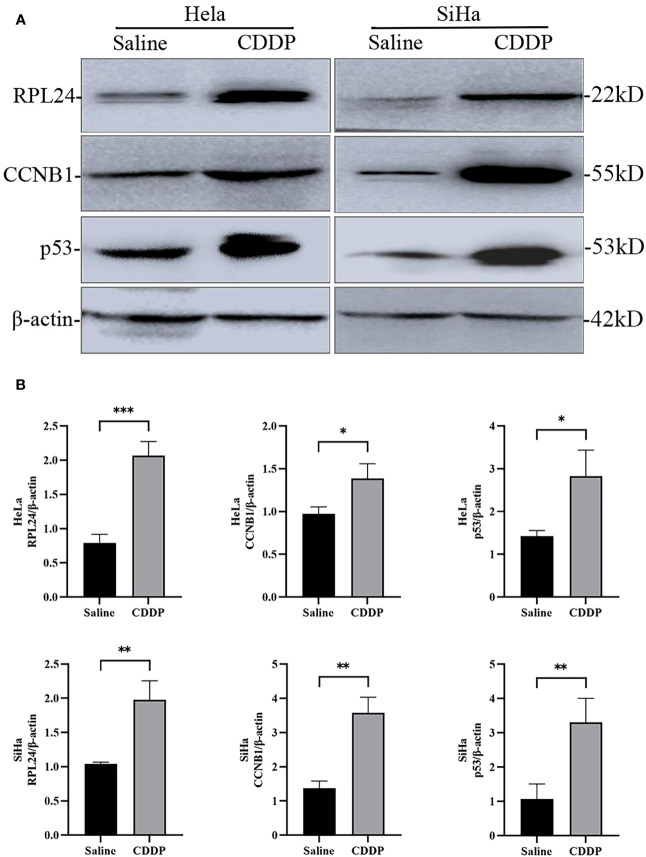
Difference in expression of RPL24/CCNB1/p53 and β-actin in SiHa and HeLa cells after saline or CDDP treatment. **(A)** Western blot analysis indicated that RPL24/CCNB1/p53 protein expression was higher in both HeLa and SiHa cells compared with before CDDP treatment. **(B)** Relative RPL24/CCNB1/p53 levels post CDDP treatment in both HeLa and SiHa cells (n=3, mean ± SD), statistical analysis was done by unpaired t-test corresponding p values shown. *r<0.05. **r<0.01. ***r<0.001.

Compared with negative control group, the proportion of G2/M phase cells was increased in HeLa and SiHa cells after CDDP treatment (**Figure 3**). The Western blotting analysis revealed, with β-actin as internal control, that RPL24, CCNB1 and p53 protein was overexpressed in SiHa and HeLa after CDDP treatment, but that it was expressed at a low level after saline treatment (**Figure 4**). CCNB1 is involved in mitotic regulation and is a marker of mitotic M-phase expression. The results of *in vitro* cell experiments showed that protein expression levels of RPL24 rose significantly in CC cell lines after CDDP treatment, accompanied by G2/M phase cells cycle arrest.

### Overexpression of RPL24 suppressed tumor growth *in vivo*

**Figure 5:**
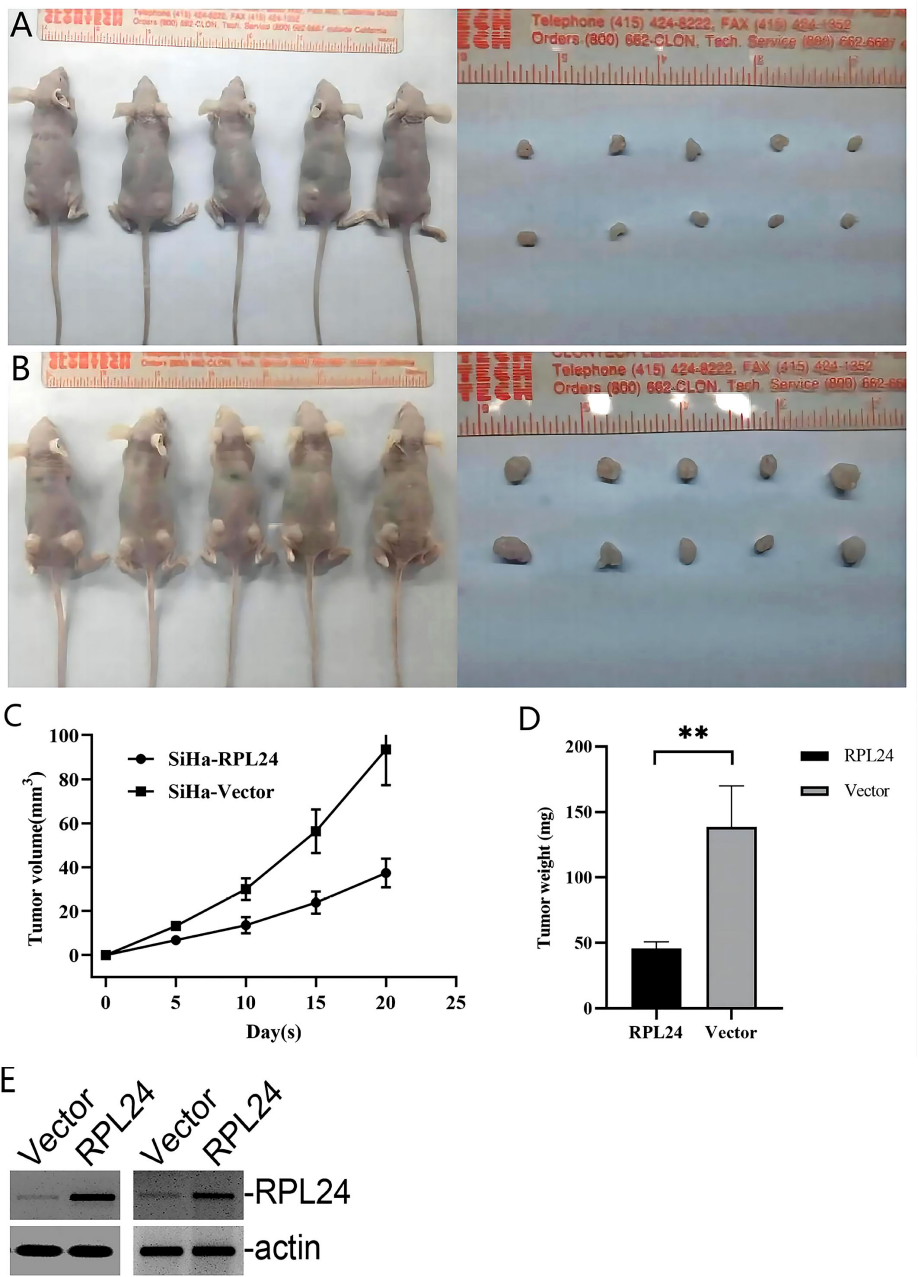
RPL24 overexpression suppresses SiHa cell proliferation *in vivo*. **(A)** Images of nude mice and xenograft tumors treated with RPL24 were captured at the end of the experiment. **(B)** Images of nude mice and xenograft tumors treated with Vector were captured at the end of the experiment. **(C)** Tumors volumes are shown as a function of days during the treatment (bars indicate the mean volume of all tumors ± SE). **(D)** Final tumor weights are shown following the treatment (bars indicate the mean weight of all tumors ± SE). **(E)** Representative immunoblots demonstrated overexpression of RPL24 in SiHa cell lines. **p<0.01.

The aforementioned results demonstrated that RPL24 inhibited CC cell development *in vitro*. Therefore, to evaluate the role of RPL24 in tumorigenesis *in vivo*, a tumor xenograft nude mice model was constructed. The mice injected with RPL24-transfected SiHa cells showed markedly better general status with a reduced rate of tumor formation (**Figures 5A, B**). The volume and weight of tumors collected after the mice were sacrificed in the RPL24 group grew slower than those of the control group (**Figures 5C, D**). The stable cell lines was identified by immunoblots that RPL24 was highly expressed in SiHa cell lines (**Figure 5E**). We showed that RPL24 overexpression can reduce the growth and tumor formation rate of the mice, indicating that the low-RPL24 expression group had a poor prognosis.

### RPL24 expression increased after CCRT in most CC patients with the better prognosis

**Figure 6:**
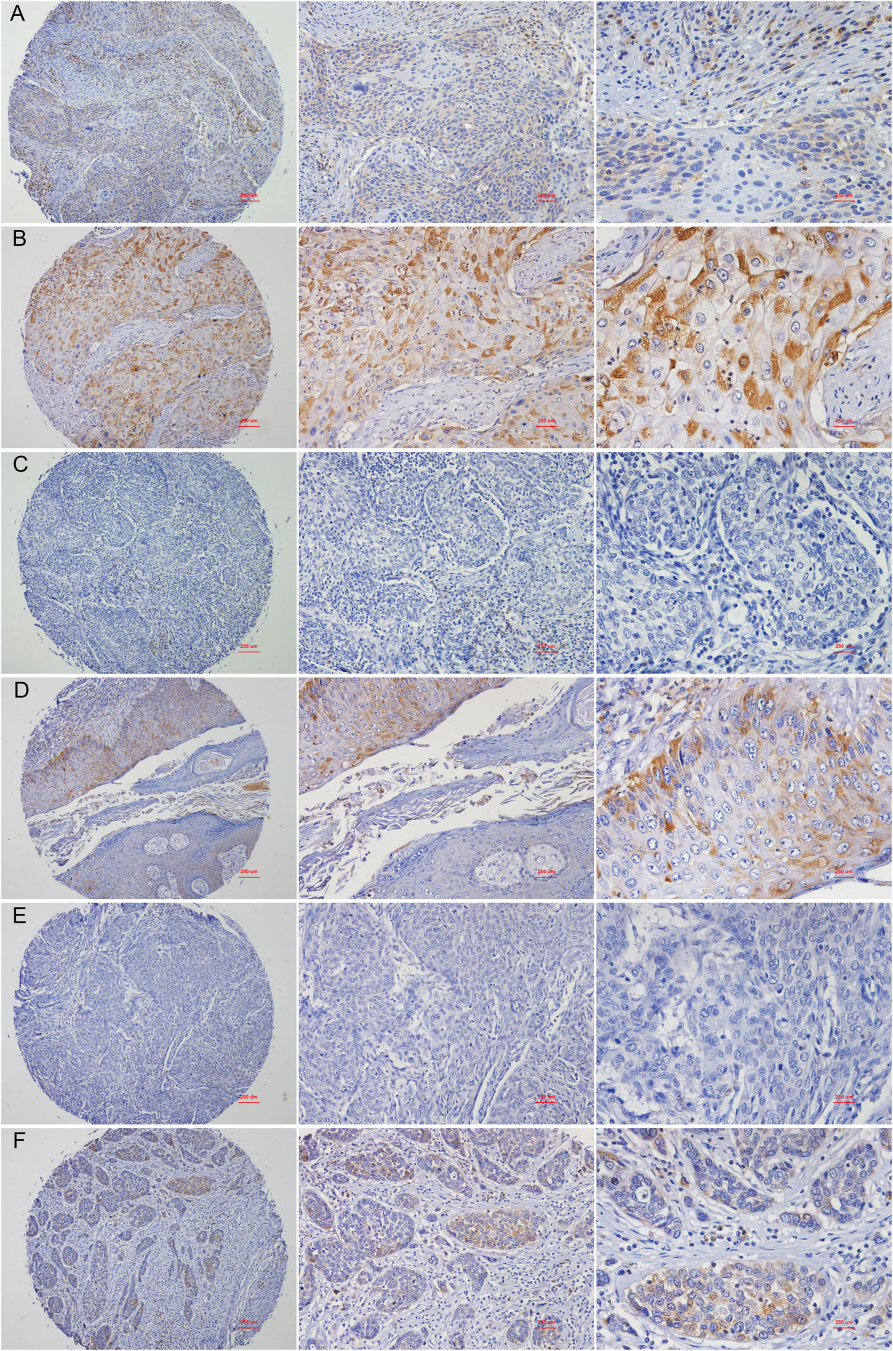
Representative images of immunohistochemical staining of RPL24. **(A)** Staining for RPL24 in CC tissues of stage of stage IB1 at ×40, ×100 and ×400 magnification respectively. **(B)** Staining for RPL24 in CC tissues of stage IB1 after CCRT at ×40, ×100 and ×400 magnification respectively. **(C)** Staining for RPL24 in CC tissues of stage IIB at ×40, ×100 and ×400 magnification respectively. **(D)** Staining for RPL24 in CC tissues of stage IIB after CCRT at ×40, ×100 and ×400 magnification respectively. **(E)** Staining for RPL24 in CC tissues of stage IV at ×40, ×100 and ×400 magnification respectively. **(F)** Staining for RPL24 in CC tissues of stage IV after CCRT at ×40, ×100 and ×400 magnification respectively.

The characteristics of the 40 patients enrolled in the study are presented in **Table 1** and representative immunohistochemical images of patients with stage IB1, IIB and IV CC before and after CCRT was shown in **Figure 6**. All patients were women with a mean age of 54.3 ± 10.0 years (range, 32 to 76). There were three cases (7.5%) of stage IB1, 34 cases (85%) of stage IIB, and 3 cases (7.5%) of stage IV CC. Twenty-six patients had a complete response (CR), 11 had a partial response (PR), and 3 had stable disease (SD). The correlation between RPL24 expression and therapeutic effect after CCRT are presented in **Table 2**. Analysis revealed that 26 CR patients and 11 PR patients highly expressed RPL24, 2 SD patients showed low expression of RPL24, and 1 SD patient showed high expression.

**Table 1:** Detailed changes in therapeutic effect, ADC value, and RPL24 expression of 40 patients with cervical cancer before and after CCRT.

S/N	Age	FIGO stage	clinical efficacy	ADC value (×10-6mm2/s)		RPL24 staining	
				Before	After	Before	After
1	58	IIb	PR	626	1030	+	++
2	41	IIb	PR	904.2	1520.6	+	++
3	43	IIb	CR	689.3	1475.2	-	+++
4	68	IIb	CR	650.4	1130	-	++
5	66	IIb	SD	775.6	1313	-	-
6	49	IIb	PR	621.6	1405	+	+
7	51	IIb	CR	673.2	1163.5	+	++
8	72	IIb	PR	736	1138.6	+	+
9	54	IIb	CR	553.7	980.3	-	+
10	62	IIb	CR	620.5	1034	+	++
11	58	IIb	SD	994	1060	-	-
12	70	IIb	CR	520.3	1034	+	+
13	62	IIb	CR	567.5	1290	+	++
14	55	IB1	CR	754.4	1210	-	++
15	32	IIb	CR	702	1106	-	++
16	56	IIb	CR	634.4	1265.6	+	+
17	49	IIb	PR	657.1	1293.5	+	+
18	44	IIb	PR	855.1	1124	-	+
19	48	IIb	CR	670	1030	+	++
20	47	IB1	PR	683.6	1311.5	-	+
21	62	IIb	CR	650	1090	+	++
22	54	IV	CR	659	1105	-	++
23	53	IIb	CR	704	1257	-	+
24	46	IV	PR	810	1200	+	+
25	76	IIb	CR	656.5	1101	-	+
26	48	IIb	CR	670.3	1069	-	+
27	50	IIb	PR	581.7	1028	+	+
28	49	IIb	CR	689	1202.9	-	++
29	45	IIb	PR	720.1	1310	-	+
30	51	IB1	CR	663	1143	-	++
31	69	IIb	CR	695.43	1179.6	+	++
32	60	IIb	CR	557.2	865	+	++
33	63	IIb	CR	651	1097	-	++
34	42	IIb	CR	698	1098	-	+++
35	39	IIb	CR	748	1203	+	+
36	47	IIb	CR	670	1205	-	++
37	71	IV	PR	669	1123	-	+
38	52	IIb	CR	719	1266	+	++
39	49	IIb	CR	734	1209	+	++
40	62	IIb	SD	567.5	997.3	-	-

FIGO, International Federation of Gynecology and Obstetrics; CR, complete response; PR, partial response; SD, stable disease. The result was determined by the percentage of positive stained cells and cell staining intensity as follows: negative −, weak positive +, positive ++, and strongly, positive +++.

**Table 2:** Correlation between therapeutic effect and RPL24 expression after CCRT in 40 patients with cervical cancer.

Clinical treatment		RPL24 staining after CCRT		Sum
		Low	High	
Clinical efficacy	CR	0	26	26
	PR	0	11	11
	SD	2	1	3

Pearson’s r=-0.575, p=0.004. *p<0.05.

Twenty-six patients had CR, 11 patients had PR, and 1 patient had SD in all patients with high RPL24 expression, and only 2 patients with low RPL24 expression had SD (**Table 2**). RPL24 appeared to be a marker of a good prognosis. The association between RPL24 expression and patient curative effect was significant, and a positive association between RPL24 expression and patient curative effect was also observed (r =-0.575; p=0.004). In general, patients with high RPL24 expression after CCRT had a favorable prognosis.

## Discussion

The combination of bioinformatics and medicine is current main trend and plays a significant role in identifying and predicting cancer biomarkers. Also, a large number of candidate targets can be provided for subsequent biological experimental verification, which will help promote the development of cancer precision medicine. Survival analysis is an important indicator for patients with carcinoma in assessing their prognosis. In our study, it was found that RPL24 expression was down-regulated in CC by TCGA database analysis, and based on the Kaplan-Meier plotter and LOGpc online database, a significant positive correlation between RPL24 expression status and RFS, OS, and PFS of CC patients was discovered, providing a theoretical basis for later clinical experiments, which indicated that RPL24 may play an important role in CC. To preliminarily verified the hypothesis, we used quantitative real-time PCR (qRT-PCR) and Western blotting to detect RPL24 expression in different CC cell lines, such as HCC94, C339, Hela and SiHa, and found that the expression level of RPL24 was the highest in highly differentiated CC cell line HCC94.

CDDP is well known as the first-line chemotherapy drug for CC, and it can block the cell cycle to G2/M, in which the CC cells are sensitive to the radiotherapy. The expression of p53 protein increased after treatment with CDDP, which induces p53-dependent apoptosis and p53-dependent pathways (21). RPs can interact with MDM2, inhibiting MDM2-mediated ubiquitination of p53 and regulating the transcriptional activity of p53. Importantly, RPs can enhance the ability of p53 to transcriptionally activate its target genes in response to DNA damage (22). Therefore, cells have the capability to induce G2/M cell cycle arrest.

After CDDP treatment, we found that hat protein expression levels of RPL24, CCNB1 and p53 rose significantly in CC cell lines, which was consistent with the results from KEGG enrichment analysis of coexpression gene set related to RPL24 in TCGA database, and it indicated that RPL24 may play a role in the control of CC cell cycle. It is possible that RPL24 is highly expressed in CDDP resistant cell, but we also found that CC patients with high RPL24 expression after CCRT had a favorable prognosis. By combining with the literature data, we speculated that RPL24 may be involved in the regulation of MDM2-p53 pathway and p53 monitoring system, and it played an important regulatory role in cervical carcinogenesis and development while its specific mechanisms remain to be investigated. Subsequently a mouse CC model was constructed showing that high RPL24 expression can//’/’ inhibit tumor formation rate in mice, which suggested that RPL24 has a tumor-suppressive effect in CC.

To further analyze the clinical efficacy of RPL24 on CCRT, we performed clinical research, which revealed that the expression of RPL24 increased compared with that of pre-therapy. Most women reaped benefit in terms of CR and PR, but only three showed SD. Immunochemistry demonstrated that the expression of RPL24 protein was increased in stage IB1, IIB and IV CC tissues after CCRT compared with the control group before treatment. The results showed that overexpression of RPL24 was positively correlated with the therapeutic effect. Taken together, the correlation between RPL24 expression and the prognosis of CC patients was revealed. CC patients with RPL24 expression had better prognosis and longer survival. Thus, we suggest that RPL24 may serve as a prognostic marker for CC survival and also possess potential value for CC patients undergoing CCRT. In this regard, the integration of biologic biomarkers like RPL24 with other indicators, such as those derived from FDG PET/CT imaging in cervical cancer patients (23), may provide more powerful biomarkers. This could potentially enhance future patient management decisions. similar to the research on synergies currently being investigated in other tumors (24).

However, there was a more concrete enumeration of limitations. Firstly, we observed that the expression level of RPL24 is related to p53 and cell cycle, but the exact regulatory mechanism remains unclear, which can be elucidated by other methods such as co-immunoprecipitation in future. Secondly, There was no consistency among patients at different stages who may have received different types of chemotherapy or undergone various treatment protocols prior to CCRT. Additionally, no analysis was conducted on other drugs used during CCRT in this study. So in this clinical research, confounding factors were not sufficiently controlled, which maybe potentially cause the biases in the results. In addition, the different levels of the professional experiments among the radiologist and pathologist experience also affected the results. Thirdly, we were unable to study the correlation between RPL24 and radiotherapy because of the limitations resulted from the clinical factors. Fourthly, this study only included patients with cervical squamous cell CC, so the results should not be interpreted as an extension to all CC cases. Therefore, these limitations indicate the need for a more rigorous prospective study, which includes comprehensive patient logs and initiates a study that records patient conditions during CCRT. This may uncover differences that we did not identify and provide valuable insights for the treatment and management of CC. Meanwhile, the results still need to be further confirmed in multicenter and largescale clinical trials.

## Conclusion

Cervical cancer is one of the most common gynecological tumors in women. There is a lack of specific therapeutic and prognostic relevant markers compared with other malignant tumors. Our findings provide new insights indicating that RPL24 expression levels increased after CDDP treatment, which was confirmed in human CC lines and human cervical tumor tissues, and high RPL24 expression was associated with a better prognosis, which was further verified in an *in vivo* mouse model, human cervical tumor tissues, and online databases. Therefore, RPL24 might be a potential target in the treatment and prognostication of CC.

## Data availability statement

The original contributions presented in the study are included in the article/supplementary material. Further inquiries can be directed to the corresponding authors.

## Ethics statement

The studies involving humans were approved by the Ethics Committee of Baotou Central Hospital. The studies were conducted in accordance with the local legislation and institutional requirements. The participants provided their written informed consent to participate in this study. The animal study was approved by the Ethics Committee of Baotou Central Hospital. The study was conducted in accordance with the local legislation and institutional requirements.

## Author contributions

YW contributed to conception and design of the study. XW organized the database and performed the statistical analysis. CM wrote the first draft of the manuscript. All authors contributed to the article and approved the submitted version.
